# Battle of centralized and decentralized urban stormwater networks: From redundancy perspective

**DOI:** 10.1016/j.watres.2022.118910

**Published:** 2022-08-02

**Authors:** Sina Hesarkazzazi, Amin E. Bakhshipour, Mohsen Hajibabaei, Ulrich Dittmer, Ali Haghighi, Robert Sitzenfrei

**Affiliations:** a Unit of Environmental Engineering, Institute of Infrastructure, University of Innsbruck, 6020 Innsbruck, Austria; b Department of Civil Engineering, Institute for Urban Water Management, Technical University Kaiserslautern, 67663 Kaiserslautern, Germany; c Faculty of Civil Engineering and Architecture, Shahid Chamran University of Ahvaz, 61357831351 Ahvaz, Iran

**Keywords:** Urban stormwater networks, Graph theory, Layout decentralization, Resilience, Redundancy

## Abstract

Recent research underpinned the effectiveness of topological decentralization for urban stormwater networks (USNs) during the planning stage in terms of both capital savings and resilience enhancement. However, how centralized and decentralized USNs’ structures with various degrees of redundancy (i.e., redundant water flow pathways) project resilience under functional and structural failure remains an unresolved issue. In this work, we present a systemic and generic framework to investigate the impact of adding redundant flow paths on resilience based on three strategies for optimal centralized versus decentralized USNs. Furthermore, a tailored graph-theory based measure (i.e., eigenvector centrality) is proposed to introduce redundant paths to the critical locations of USNs. The proposed framework is then applied to a real large-scale case study. The results confirm the critical role of layout decentralization under both functional (e.g., extreme precipitation events), and structural failure (e.g., pipe collapse). Moreover, the findings indicate that the implementation of redundant paths could increase resilience performance by up to 8% under functional failure without changing the network’s major structural characteristics (i.e., sewer diameters, lengths, and storage capacity), only by leveraging the effective flow redistribution. The scheme proposed in this study can be a fruitful initiative for further improving the USNs’ resilience during both planning and rehabilitation stages.

## 1 Introduction

Urban stormwater networks (USNs) have a focal role within urban water ecosystem in preventing pluvial flooding in urban areas. Achieving a high level of reliability to minimize the frequency and magnitude of failures is the conventional approach to designing USNs (David Butler et al., 2017). This approach has left communities with vulnerable infrastructures to increasing threats such as natural disasters and climate change (Sweetapple et al., 2022). In addition, a detailed assessment of planning, rehabilitation, and management of urban water infrastructures alongside their modeling is often not trivial due to high complexity and interconnection between their components (Hajibabaei et al., 2019). Such hurdles are more exacerbated when accounting for the unprecedented impact of climate change combined with ever-increasing urbanization (Butler et al., 2014; Casal-Campos et al., 2018; Sitzenfrei et al., 2022; Webber et al., 2022). These deviations often manifest themselves in heatwaves, flooding and droughts plaguing cities across the globe (AghaKouchak et al., 2014; da Silva et al., 2022; Hesarkazzazi et al., 2021; Najafi et al., 2021). For this reason, it is necessary to learn to adapt the infrastructures to mitigate the effects of such exceptional conditions (Shukla et al., 2019); and therefore, a paradigm shift from reliability to resilience for urban water infrastructures during all stages including planning, operation, and rehabilitation is needed (Sweetapple et al., 2022). Recent discussions also regard resilience as the core of sustainable development where systems must be resilient to overcome future uncertain threats (Juan-Garcia et al., 2017).

In this context, various definitions and interpretations surrounding resilience have been documented in recent literature (Dong et al., 2017; Leandro et al., 2020; Mugume and Butler, 2017; Wang et al., 2021, 2019). However, in this paper, we adopt the concept introduced in Butler et al. (2014); and Mugume and Butler (2017), wherein resilience is defined as “the degree to which the system minimizes level of service failure magnitude and duration over its design life when subject to exceptional loading conditions”. Some literature also divides resilience into two broad categories, specified and general resilience (Sweetapple et al., 2022). Specified resilience is the “resilience of some particular parts of a system... to one or more identified kinds of shocks,” whereas general resilience is the “resilience of any and all parts of a system to all kinds of shocks, including novel ones” (Folke et al., 2010). As it is impossible to predict all potential threats, building general resilience in USNs to minimize the magnitude and duration of failure when such unexpected threats occur is crucial (Sweetapple et al., 2022). In relation to the threats jeopardizing USNs, there are two common types of failure: (1) functional failure caused by the load alterations on the system, and (2) structural failure caused by issues that disrupt the function of single or multiple components of the system (Mugume et al., 2015). The former (functional failure) is induced by climate-change-driven precipitation or urbanization increases (Butler et al., 2018), and the latter (structural failure) is induced by components’ failure such as sewer blockage, pump failure and sensor failure (Sweetapple et al., 2022). Thus, to build general resilience in USNs, both functional and structural failures must be considered (Guptha et al., 2021; Osheen et al., 2022)

The first step in achieving an appropriate level of resilient performance is planning and designing of a USN comprising of a proper spatial layout and hydraulic design. Most studies regarding urban drainage planning have focused on hydraulic design and sewer sizing strategies to achieve optimal solutions (Afshar and Rohani, 2012; Ahmadi et al., 2018; Diao, 2020; Karovic and Mays, 2014). A few studies have proposed creating optimal topological layouts as the foregoing step, among which the most commonly presented methodology adopts the generation of centralized topological layouts (Duque et al., 2020; Haghighi and Bakhshipour, 2012; Hesarkazzazi et al., 2021; Hsie et al., 2019; Moeiniand Afshar, 2017). However, these reliability-based optimization methods often minimize construction/design costs, and consequently, lessen system resilience. To overcome this challenge, recent research has emphasized the effectiveness of topological decentralization at the planning stage in terms of both capital investment and resilience enhancement (Bakhshipour et al., 2019; Björklund et al., 2018; Eggimann et al., 2015; Hesarkazzazi et al., 2022; Wang et al., 2021; Xu et al., 2021). Consequently, topological decentralization at the design stage for improving system resilience is paramount, which needs to be undertaken first. However, for existing USNs, a transition from centralized to decentralized systems in most cases is unattainable or at least too problematic. As a consequence, we need to consider other alternatives to build resilience in these existing USNs. Despite certain variations and modifications in the literature on resilience-promoting attributes within the realm of USNs (Kwon et al., 2021; Wang et al., 2019), we adhere to two broad categories introduced by Mugume et al. (2015): (1) flexibility, and (2) redundancy. Flexibility is defined based on the inherent system’s ability to be reconfigured and adjusted to maintain an acceptable performance level under multiple (varying) loading conditions. Flexibility can be implemented via distributed/decentralized elements such as green-blue infrastructures (GBIs), e.g., implementation of green roofs, rain barrels, permeable pavements, etc. across the catchment. However, redundancy attribute implies having multiple components with similar functions to lessen failure propagation through the entire system. Redundancy can be enriched in practice by presenting additional storage tanks, providing more spare capacity at certain critical locations (e. g., enlarging pipes), and adding alternative water flow paths (forming loops), which (last one) is the focus of this study.

To elaborate more on these attributes, for instance, GBIs can play a crucial role in enhancing the resilience of USNs. In contrast to grey infrastructures, GBIs are more flexible in terms of installation, adaptation, bringing multifunctions and co-benefits to urban stormwater management (Sitzenfrei et al., 2022). In recent literature, the benefits of these kinds of adaptation measures have been widely discussed and accepted (Bakhshipour et al., 2021; Bakhshipour et al., 2019; Haghighatafshar et al., 2018; Liu et al., 2021; Oberascher et al., 2022; Pour et al., 2020; Wang et al., 2022). For example, Wang et al. (2022) proposed a framework for designing hybrid green-blue and grey USNs considering both technological and operational resilience. They defined technological resilience as the system’s performance under extreme loads and operational resilience as the performance and long-term efficiency of the system after structural damage or degradation. McClymont et al. (2020) proposed a resilience-driven multi-objective optimization model to find the optimal level of the spatial distribution of GBIs based on water quality and quantity performance. Recently, smart data-driven measures and decentralized real-time control options have been frequently introduced and addressed to enhance urban water systems’ resilience (Sitzenfrei et al., 2022). For instance, Oberascher et al. (2022) implemented smart rainwater harvesting to reduce potable water consumption and improve urban drainage performance and further evaluated the resilience of the integrated system on a large-scale implementation. These findings show that the most resilience-boosting solutions are costly but yet do not guarantee the higher quality of life index values. To conclude, building resilience through conventional approaches (e.g., by presenting additional storage or enlarging sewers’ diameter) or more sophisticated methods (e.g., GBI and smart control) might be effective but expensive. Besides, resilience is often seen as an extra expense or additional effort in urban water systems planning (Juan-Garcia et al., 2017). Moreover, introducing components for enhancing resilience to the design, retrofit, or rehabilitation procedure of USNs increases the complexity of the design/operation problem exponentially. Thus, instead of a single threat, various threats and their combinations must be considered (Bakhshipour et al., 2021), and as mentioned before, since it is impossible to predict all potential threats, building general resilience in USNs is highly recommended (Sweetapple et al., 2022).

As USNs are often composed of gravity-driven pipelines with a tree-like structure, incorporating loops is not a common practice in these infrastructures. This has culminated in a very small body of research reporting the consequences of adopting alternative water flow paths (forming loops) as a cost-effective approach for resilience enhancement in USNs. Since all sewers (edges of the base graph) must be included in the final design of a USN (each of them drains a certain street Haghighi, 2012), implementing redundant flow paths through this cost-effective way to build general resilience may be an efficient adaptation or design measure. Hereafter, alternative/redundant water paths (or loops) are interchangeably referred to as redundancy. In light of this, some studies proposed frameworks to describe the topological characteristics of drainage networks. For example, Meshness coefficient proposed by Reyes-Silva et al. (2020) leverages graph-theory-based concepts to quantify and compare the networks with various levels of existing redundant paths with tree-like and grid-like (loop-shaped) networks. The drainage density index suggested by Lee et al. (2018) is also used to evaluate the structure of the drainage pipe network as the ratio of total pipe length to catchment area, indicating the degree of pipe network density. When it comes to the consequences of adopting redundancy for structural failures, Yazdi (2018) and Zhang et al. (2017) explored the influence of redundant sewers on vulnerability and resilience of USNs, respectively, under pipe blockages. They reported that the loop-shaped structures demonstrate less vulnerable components and higher resilience than tree-like ones under such failure type. However, concerning the implications for functional failure, Seo et al., (2015) applied the Gibbs’ stochastic network model (Troutman and Karlinger, 1992) to create and compare the performance of dendritic with fully looped networks. They inferred that the shape of the hydrograph and the fully looped system’s peak flow are highly correlated with the catchment slope. Lu et al. (2021) explored the impact of dendritic and looped drainage networks in two catchments in China. They concluded that the loop-shaped network is more likely to reduce peak runoff at the outlet for the purpose of flood risk reduction. Reyes-Silva et al. (2020, 2021) reported that under functional failure, loop-shaped USNs provide a more resilient response (e.g., recording less flood volume and flood damage costs) than tree-shaped ones. Hesarkazzazi et al. (2020) proposed an innovative approach that leverages complex network analysis to eliminate alternative water paths from upstream and downstream of a fully looped USN to infer resilience variations. However, certain deficiencies hinder the generalization. For example, they have resorted to invert elevations of the inlets/manholes to determine the removal of sewers from upstream and downstream. Nonetheless, invert elevations may not always yield an accurate indication of upstream/downstream because it primarily depends on the network design characteristics. Finally, the impact of the structures (possessing loops) was addressed in Hesarkazzazi et al. (2020) at the design stage only, meaning that the networks with various numbers of loops were simultaneously designed and then evaluated. There also exists an example of how redundant flow paths (in looped networks) are capable of reducing the frequency and magnitude of combined sewer overflows (CSOs) in a catchment in Dresden, Germany (Reyes-Silva et al., 2020). However, the few aforementioned studies did not address the mechanisms by which redundant paths can improve the resilience of different USN structures (centralized versus decentralized) under both functional and structural failures. Moreover, currently there is no guidance on the optimal placement of redundant paths for maximizing resilience at the design or rehabilitation stages for existing USNs.

To conclude and based on the presented literature review, we identified the following research needs:

- Developing cost-effective and easy-to-implement methods to build resilience in USNs as an alternative to existing expensive approaches such as adding GBIs or extra storage capacity.
- Developing a computationally efficient framework for including and assessing redundant flow pathways (forming loops) in optimal planning and/or rehabilitation of (de)centralized USNs.
- Benchmarking the performance of the proposed methods using real-world test cases.

Therefore, this study aims to bridge these research gaps, whose key contributions are as follows:

- Developing a graph-theory-based and computationally inexpensive method to optimally introduce redundancy by leveraging an effective flow redistribution throughout USNs with practical guidelines for implementation in the real world.
- Proposing a framework to systematically assess the impact of redundancy on resilience for optimally designed (de)centralized USNs, experiencing functional and structural failures.
- Applying the proposed methods and frameworks to a large-scale real case study to discover the importance of topological decentralization in relation to redundancy.

The remainder of the manuscript is structured as follows. Section 2 introduces the relevant methodologies and case study utilized in this study. Section 3 discusses the implications of the developed methodology for functional and structural failures. Section 4 outlines the limitations, advantages and future work, and finally Section 5 summarizes the key takeaways from this study.

## 2 Methods

Section 2.1 provides an introduction to graph theory analysis and the measures applied in this study; Sections 2.2 and 2.3 discuss the (de) centralized layout generation procedure along with their hydraulic design optimization; and Sections 2.4-2.6 discuss redundancy-promoting strategies, resilience assessment, and suggested case studies, respectively.

### 2.1 Complex network analysis of USNs

The USN topology can be mathematically represented and analyzed using graph theory. Consider a graph G consisting of a set of vertices V (e.g., manholes, storage units, and outlets) interconnected by a set of links or edges E (e.g., conduits, weirs and pumps). A convenient method to represent graphs is to use an adjacency matrix. The adjacency matrix of a graph is matrix A whose entry *a_ij_* = 1 if there exists an edge (*i*, *j*), and *a_ij_* = 0 otherwise. If the directions of the edges are considered in the graph, it is referred to as a directed graph (in contrast to an undirected graph). In addition, various weighting functions for the graph edges, such as sewer diameters, lengths, and hydraulic properties can be defined.

The first graph measure utilized in this study is the shortest path σ_*i,j*_ which is the path between nodes *i* and *j*, wherein sum of (positive) edge weights is minimal (Dijkstra, 1959). The shortest path can be determined for directed and undirected graphs. During the design (layout generating)/rehabilitation phases (see Sections 2.2 and 2.4), the shortest path algorithm is applied by considering the Euclidean distance between node pairs (i.e., sewer lengths) as weighting functions. Another definition utilized in this study is the node degree (degree centrality), which indicates the number of edges connected to a node. The more connections a node has, the higher the importance (degree centrality) is. In directed graphs (e.g., directions based on flow directions or ground elevations), the total node degree $\left(k_{i}^{tot}\right)$ has two components: (1) node indegree ($k_{i}^{in}$) representing the number of incoming edges to a node, and (2) node outdegree ($k_{i}^{out}$) representing the number of outgoing edges from a node, shown in Eq. (1).

(1) $$k_{i}^{tot}=k_{i}^{in}+k_{i}^{out}$$

where *a_ij_* represents the entries of the adjacency matrix.

The next measure in this study is the eigenvector centrality utilized for scoring and ranking influential nodes/junctions for the introduction of redundant paths. Eigenvector centrality is a method of generalizing the degree of centrality, which premises that a node’s importance can increase in the network by having connections to the other nodes which themselves are important. The eigenvector centrality of a node is computed by assigning each node a relative score proportional to the sum of the scores of its connections. Therefore, the scores are normalized by the largest eigenvalue of the nodes. The eigenvector centrality of a node is attained a large value if the node has many connections and/or many important connections Bonacich, 1972; Latora et al., 2017; Rajagopal et al., 2017). To model the USN characteristics, we assigned sewer diameters to edge importance/weight of the graph. This considers the number of adjacent connections to a node and/or their importance relative to the sewer diameters. Therefore, it allows the identification of the nodes that directly and indirectly interact and connect to the main collectors of the network (with large diameters). The nodes are then ranked based on their eigenvector centrality scores to introduce redundancy. The higher the ranking, the higher the priority given to that node to construct a redundant path (see Section 2.4). Eqs. (2) and ((3) represent the eigenvector centrality measure:

(2) $$\lambda c_{i}=\sum_{j=1}^{\#V}a_{ij}\;c_{j}$$

where, *i* = 1, 2, …., *V*, are the nodes, *λ* is the largest eigenvalue of A, *c_i_* is the eigenvector centrality (eigenvalue) of node *i*, *c_j_* is the eigenvector centrality (eigenvalue) of other neighboring nodes relative to node *i*, and *a_ij_* are the entries of adjacency matrix A of undirected graph. In matrix notion, Eq. (2) can be written as follows:

(3) $$Ac^{E}=\lambda c^{E}$$

where *c* is an N-dimensional column vector (eigenvector) whose entry is equal to *c_j_*.

**Fig. 1:**
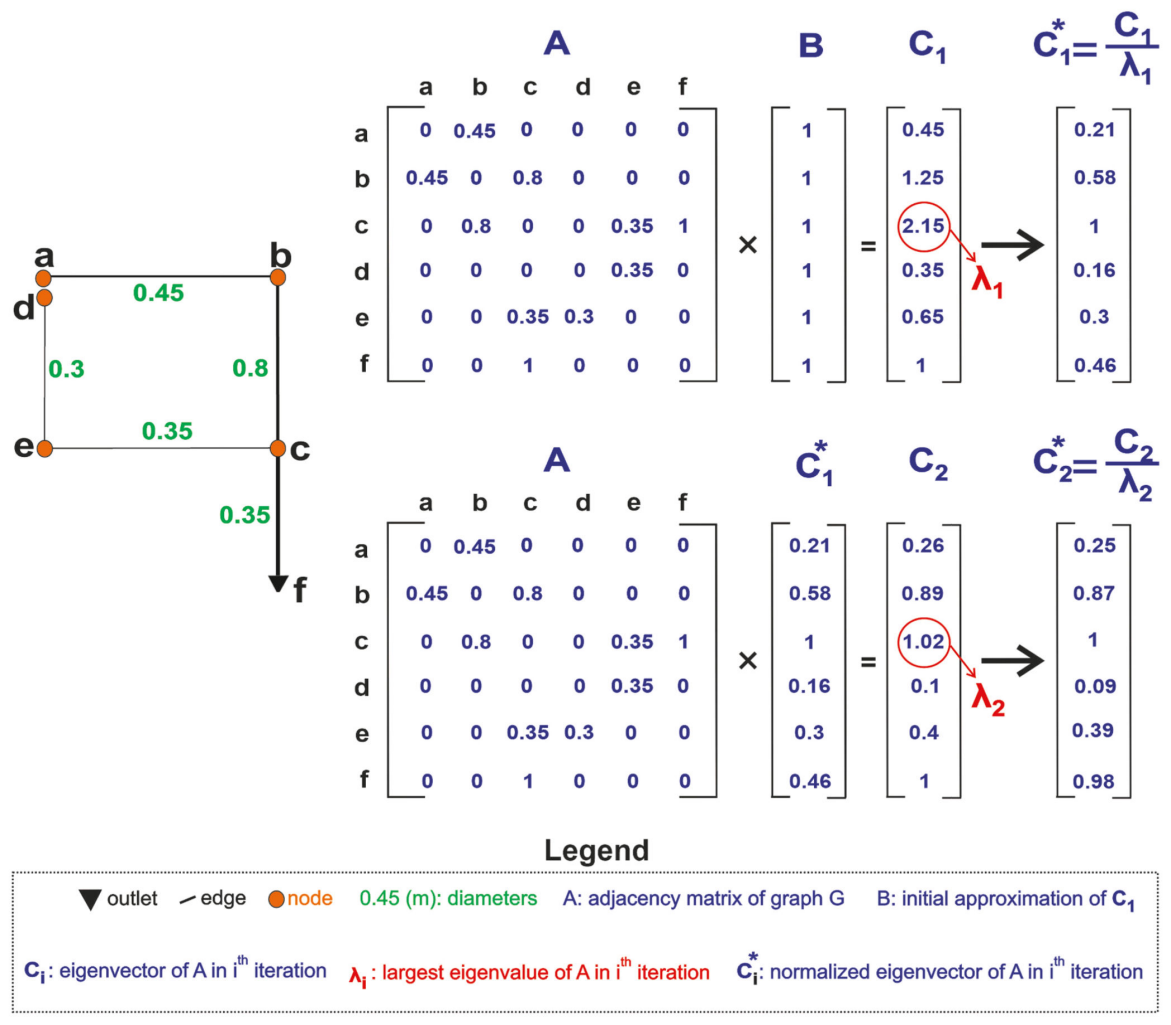
Illustrative example of how eigenvalues and eigenvector are calculated.

An illustrative example of how eigenvalues and eigenvector are calculated using the power method algorithm for a simple graph below is shown in Fig. 1. Note that this procedure is an iterative approach.

First, the adjacency matrix of the graph with pipe diameters as edge weighting function is calculated (A). Then, in the first iteration, this matrix is multiplied with an initial approximation vector (B), where we assigned all values to 1. The result is the eigenvector of A in the first iteration (*C*_1_), whose largest value (*λ*_1_) is selected. This value is then used to normalize *C*_1_, yielding the normalized eigenvector in the first iteration $C_{1}^{*}$. In the second iteration, A is multiplied with the calculated $C_{1}^{*}$ to obtain the eigenvector in the second iteration (*C*_2_). Again, the largest eigenvalue in the second iteration (*λ*_2_) is selected to normalize *C*_2_, resulting in $C_{2}^{*}$. This procedure is continued until all eigenvalues become stable, and finally yielding the stable version of eigenvector of A. At the end, one can normalize the eigenvector values one more time so that the sum of all nodes’ centrality (values) is equal to 1.

### 2.2 (De)centralized layout generator

**Fig. 2:**
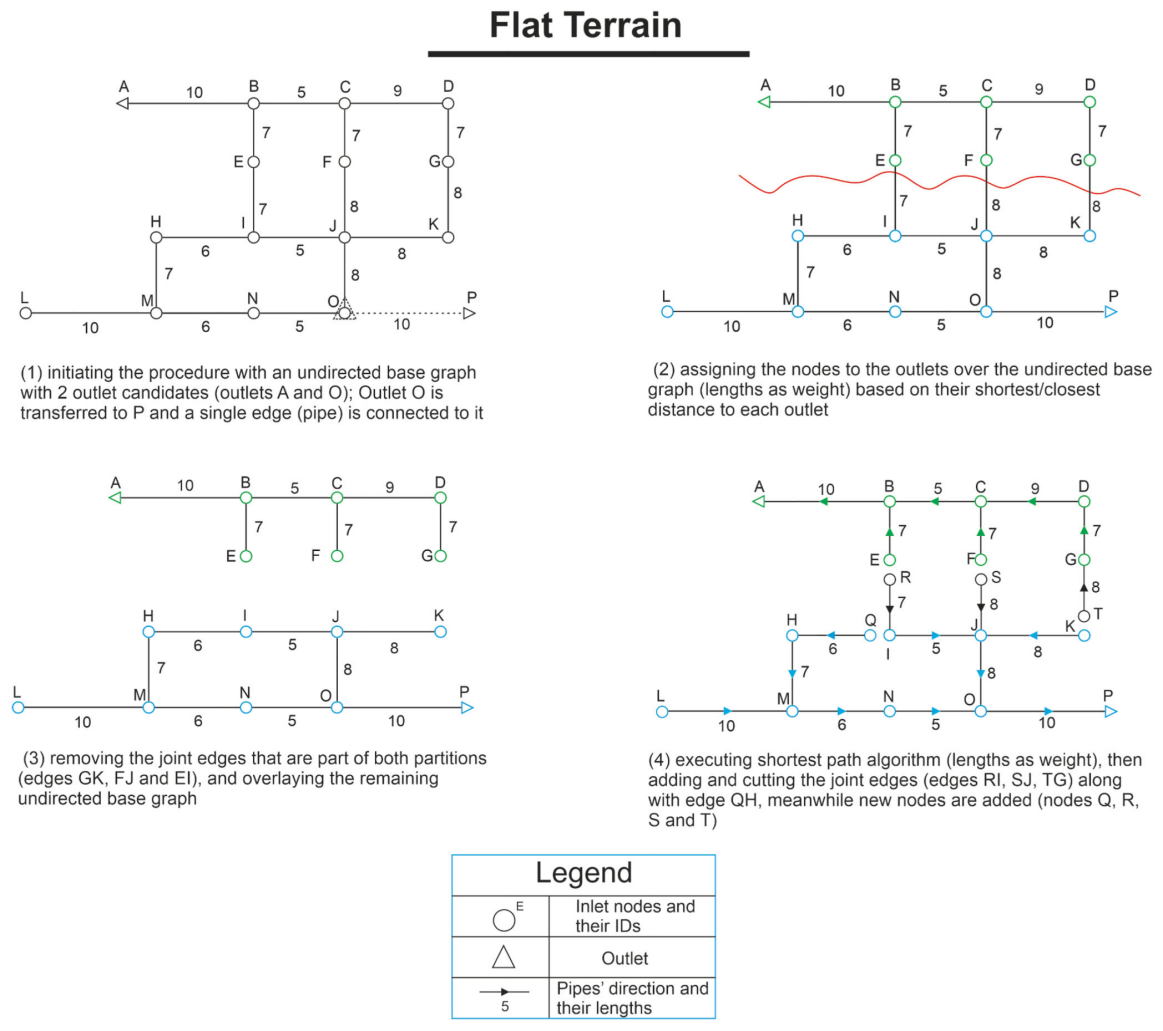
Layout generator module for flat terrain areas (Hesarkazzazi et al., 2022).

The layout of a USN, representing the arrangements and configurations of sewers and junctions/manholes along the sewer flow directions, has a critical role in achieving an optimal and low-cost design (Bakhshipour et al., 2019; Hesarkazzazi et al., 2022; Zhang et al., 2020). Given an entirely flat area (i.e., no effective ground slope as well as directions exist at the planning stage), the layout generation process is executed over the undirected base graph (with sewer lengths as edge weighting function). The base (initial) graph represents the network (i.e., street network of the desired area), which contains all possible sewer pipelines (all drainage possibilities), upon which the layout generation process (flow directions determination) is initiated. The reason why street network is adopted as the initial base graph is the strong correlation between sewer and road networks. For instance, Mair et al. (2017) concluded that, on average, 50% of the street network lengths correlates with 85% of the total sewer network. The step-by-step procedure of decentralized layout generation is illustrated and explained in Fig. 2 when the location of outlets is defined. Note that the process described in this Figure is derived from a bigger framework explained in Hesarkazzazi et al. (2022), wherein layout creations for flat as well as steep areas were addressed separately. Therefore, since a flat case study is considered in this study, we only selected and explained the relevant methodology for flat regions.

It should also be noted that when only a centralized solution is required (one outlet is assigned), a similar procedure outlined in Fig. 2 for each separated branch (subgraph) can be applied (see Steps 3 and 4). This implies that a simple shortest path algorithm from all (inlet) nodes to the outlet node is performed over the initial undirected base graph (with pipe lengths as graph weights) to attain the pipe directions. Then, the removed edges/pipes during the shortest path execution are added and cut to generate a tree centralized network that contains all the sewers (see Steps 3 and 4 in Fig. 2).

However, assigning a set of pre-defined outlets (as outlet candidates) to the specific locations of base graph (e.g., in the proximity of river stream), necessitates the exploration of all possible layout configurations of outlet placements (i.e., different degree of centralization). The degree of centralization (*DC*) implicitly quantifies how the USN as a whole is distributed (Hesarkazzazi et al., 2022) as outlined in Eq. (4). This measure builds a logarithmic relationship between the number of (selected) outlet nodes (*ON_s_*) from a list of possible outlet candidates, and total number of inlet nodes (*IN*):

(4) $$DC=100\times \left(1=\frac{log_{10}^{ON_{s}}}{log_{10}^{ON}}\right)\left(\%\right)$$

This measure denotes that if all drainage inlet nodes act as outlet nodes, *DC* is computed equal to 0% (complete decentralization), which is of course unfeasible. Whereas, if only one outlet node is selected among all the outlet candidates, *DC* is computed equal to 100% (fully centralization). As demonstrated in Fig. 2, the developed layout generation process herein is a deterministic approach, implying that only one unique layout solution is created for each outlet placement scenario. Hence, when a set of pre-defined outlet candidates are defined and placed, as previously mentioned, all possible combinations of outlet placements should be enumerated (different *DCs*). This is done to finally uncover the optimal locations and numbers of outlets from that set of pre-defined outlet candidates (i.e., finally selecting the optimal network). However, hydraulically designing all generated layouts to achieve the optimal one culminates in a significantly large computational demand. Therefore, a combinatorial optimization framework (i.e., brute force or exhaustive search) with two layout objective functions was used, wherein the only decision variable is the number of possible outlet candidates (*N_PO_*) with 2^*Npo*^ - 1 possible solutions. These two layout objective functions aim to pre-screen and discard a large number of the generated layouts prior to hydraulic design optimization. These layout functions operate based on sewer lengths and runoff areas distribution through the sewers imposed during the layout generation in order to find the networks having the efficient distribution, and subsequently, minimizing the hydraulic design costs. Then, all the layout solutions lying on the Pareto-front (as a result of interaction of the two layout objective functions) are chosen and forwarded to the hydraulic design optimization (see Section 2.3.) to ultimately choose the optimal fully designed network (with the smallest design cost). Further details regarding the layout generation process can be found in Hesarkazzazi et al. (2022).

### 2.3 Hydraulic design optimization

As discussed above, the layout solutions on the Pareto-front are all forwarded to hydraulic design optimization. This is performed to size the sewers, slopes and pump stations while a set of design criteria objectives and constraints are satisfied. To achieve this goal, the hydraulic design algorithm introduced by Haghighi and Bakhshipour (2012) was adopted in this study using an adaptive approach. The dynamic wave flow equations in the SWMM (Rossman and Huber, 2016) were used to design all networks. The algorithm requires 3NP decision variables given NP is the number of pipes/sewers. This includes NP discrete variables for sewer sizes, NP real variables for sewer slopes, and NP binary (0-1) variables to determine whether there should be a pump station at a sewer upstream. Furthermore, life cycle cost (LCC) is considered for the design costs of the case study. LCC (dos Santos et al., 2021) includes the capital, operation, and maintenance costs for a 30-year service life. As a result, the minimization of LCC is set as the only objective function of the hydraulic design optimization framework. Surcharge has been permitted while no flooding is allowed to occur in the system under design storm event. A 2-year block rain event was utilized for the pipe sizing process according to Iranian standards and regulations in flat areas. Layouts that require lift stations are automatically omitted using a penalty function during the optimization. In this study, as the area is completely flat, the minimum allowable slopes from the local manual are allocated to each pipe based on their diameters. Therefore, the problem simplifies to finding the optimum diameter of pipes in the generated layouts to minimize LCC. Thus, in adaptive algorithm only one vector of decision variables (*P*) with size NP is needed to assign the diameter of pipes using Eq. 5 as follows:

(5) $$D=D_{min}+\left(D_{max}-D_{min}\right)\times P$$

where *D_max_* is the largest commercially available size and *D_min_* is determined with respect to the telescopic pattern. The diameter of every pipe must be equal to or greater than that of its upstream pipes, which means that:

(6) D≥max[DU]

where [*DU*] contains pipe diameters connected to the upstream end of the pipe at hand.

Within our optimization framework, we employed a genetic algorithm as the optimization engine to find the optimal values for decision variables *P*. The other considered constraints included the minimum and maximum cover depths, maximum slopes, and maximum velocity, all of which were chosen based on the regulations and standards for the analyzed city. Once all the solutions in the Pareto-front are hydraulically designed, the optimal (decentralized) layout solution is chosen and proceeded to the redundancy deployment process (see Section 2.4.). Note that all the centralized configurations (having different outlet locations) are also hydraulically designed, among which the best one (with cheapest LCC) is selected to compare the characteristics of redundancy between centralized and decentralized (optimal) layouts. Further details regarding the hydraulic design optimization framework, design constraints and LCC equation can be found in this study’s Supplementary Material.

### 2.4 Redundancy-promoting strategies

Once the optimal (decentralized) network as well as the centralized one were chosen and hydraulically designed, we established a framework to build resilience using a specific redundancy attribute (i.e., adding additional water pathways/redundant paths). This can be performed during the design or rehabilitation stage for existing systems. Specifically, we are interested in how the flow (re)distribution, imposed by different numbers and locations of redundant flow paths within the optimal (de)centralized networks, affects the resilience.

**Fig. 3:**
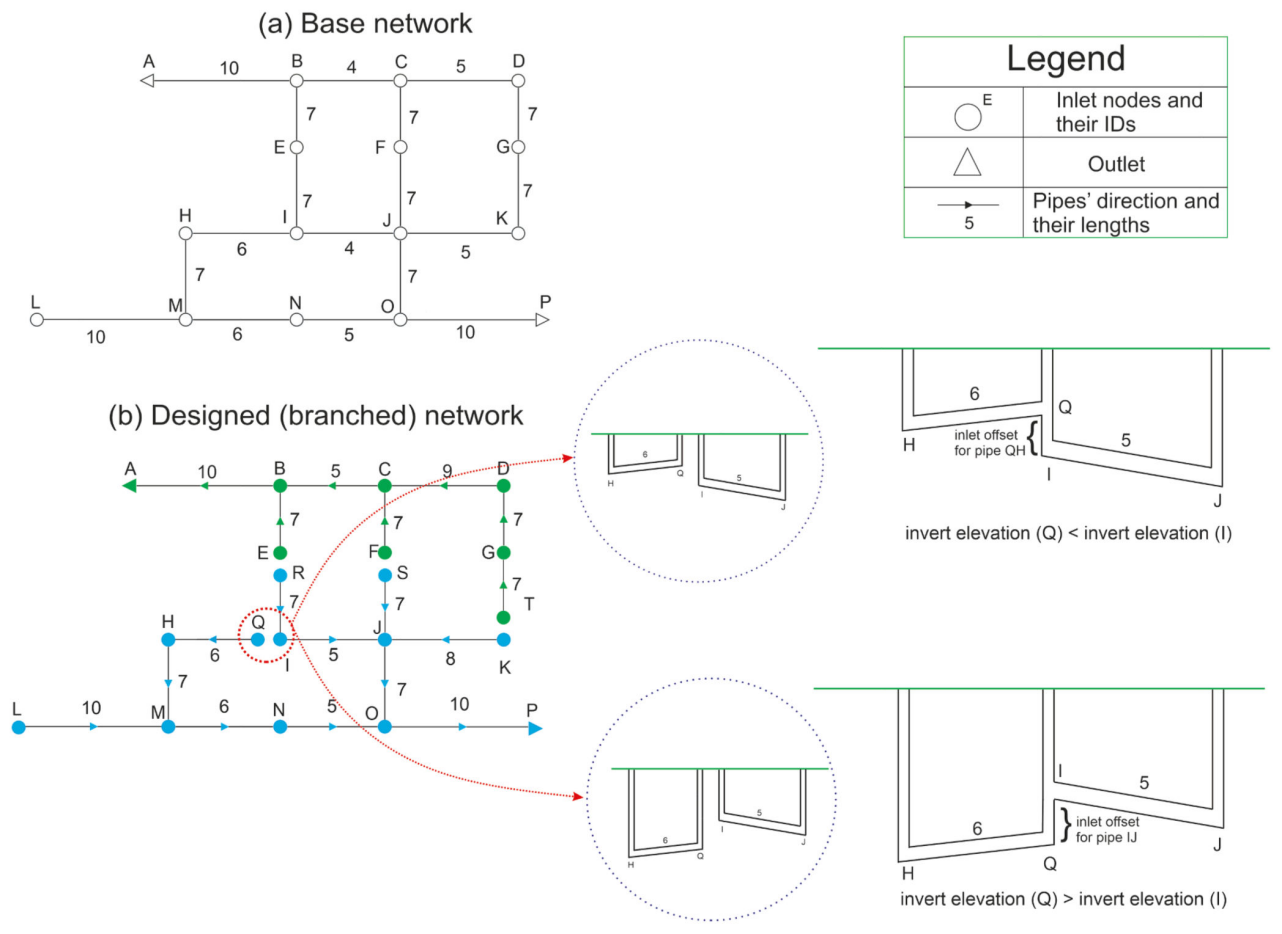
Pedagogical example of redundancy placement.

The candidate nodes to construct redundant paths are those added (after the edges/sewers of the base graph are cut) to the network during the layout design procedure prescribed in Section 2.2. This approach is applicable to both centralized and decentralized systems. However, pre-screening of some of these candidate nodes should be performed beforehand for a decentralized system. For instance, as shown in Fig. 3, nodes T, S, R, and Q are potential candidates for adding extra paths within the networks by comparing the base graph (Fig. 3(a)) with the final designed graph (Fig. 3(b)). However, connecting nodes T, S, and R violates the decentralization notion of its structure, and thus, couple the two separated subgraphs. As we tend to uncover the characteristics of redundancy attributes for each structure (centralized vs. decentralized networks) separately, the nodes T, S, and R are excluded from the redundancy candidates, and we continue working with node Q only. Subsequently, to add the path to the network concerning node Q, depending on the achieved design characteristics for node Q and its adjacent one (node I), two situations may occur (Fig. 3):

(1) Invert elevation of node Q is smaller than the invert elevation of node I.

If this scenario occurs, the sewer QH is connected to manhole I with a new inlet offset equal to the difference between the Q and I invert elevations.

(2) Invert elevation of node Q is larger than the invert elevation of node I.

If this situation occurs, the sewer QH is connected to manhole I while a new inlet offset equal to the difference between the Q and I invert elevations is placed for sewer IJ.

This procedure is repeated for all candidate nodes one by one (i.e., alternative pathways are added cumulatively and consecutively) for decentralized network to finally achieve the network with the maximum number of redundant paths (inner loops within each subgraph). For centralized network, there is, of course, no pre-selection of the candidate nodes, and all the nodes cut from the base graph during the layout generation stage are considered for the introduction of redundant paths.

Alternative water flow pathways in this study are systematically and consecutively implemented based on three scenarios/approaches:

(1) Adding alternative paths from the upstream sections towards downstream sections (hereafter referred to as the upstream scenario/approach).
(2) Adding alternative paths from the downstream sections towards upstream sections (hereafter referred to as the downstream scenario/approach).
(3) Adding alternative paths starting from certain locations determined by eigenvector centrality (hereafter referred to as the centrality scenario/approach).

**Fig. 4:**
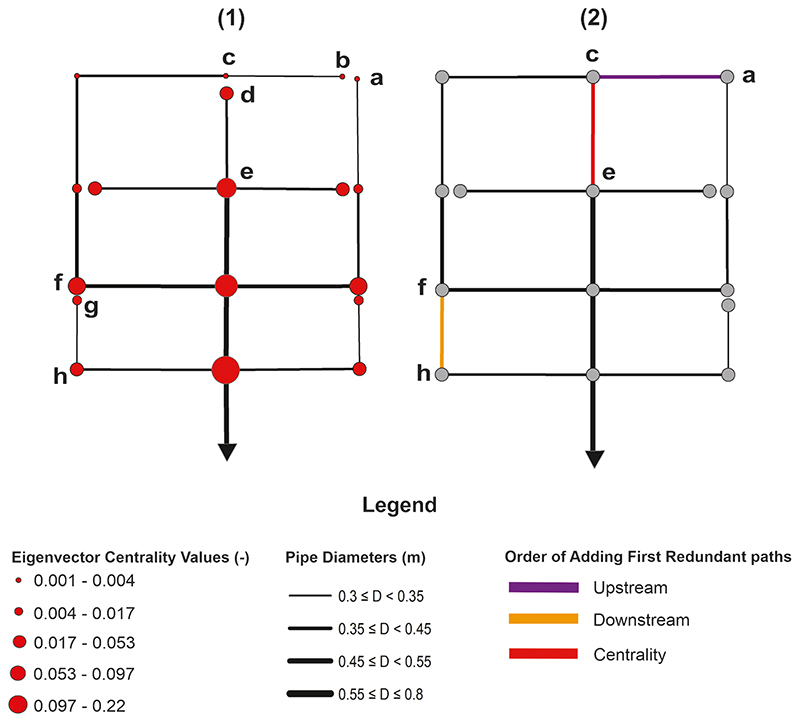
Pedagogical example for (1) centrality approach (centrality rankings for the graph); and (2) order of how the first redundant flow path is constructed based on three approaches (upstream, downstream and centrality).

To determine the upstream and downstream locations in scenarios (1) and (2), the shortest path algorithm was executed over undirected branch graphs (i.e., designed centralized and decentralized networks) with sewer lengths as edge weights. The undirected graph (with lengths as edge weights) is used to determine the Euclidean distance of each node to its corresponding outlet using the shortest path algorithm, which has not been feasible in a directed graph as a consequence of direction interference. This method yields a more accurate indication of upstream or downstream nodes. The shorter the distance of a node from its corresponding outlet, the more downstream the location. However, scenario (3) seeks to cumulatively add alternative paths from the most influential nodes determined by eigenvector centrality using Eqs. (2) and (3). To clarify more on this approach and how additional paths are constructed, a pedagogical example is provided in Fig. 4 below, wherein (1) shows the eigenvector centrality rankings for the nodes (using the sewer diameters as edge weights of the graph); the bigger the red-coloured nodes, the larger the centrality values, and (2) shows the order of adding the first redundant path based on the aforementioned three approaches (upstream, downstream and centrality). The first redundant path using the upstream and downstream approaches are edges/pipes ac (pink colour) and fh (orange colour), respectively as shown in (2). Using the eigenvector values computed in (1), from all the nodes, cut from the base graph, node d has the largest eigenvalue. Therefore, the first path based on the centrality approach is constructed as edge/pipe ce (red colour) shown in (2).

It is important to note that a minor modification is added to scenario (3) or centrality approach regarding structural failure implications in Section 3.2. Nine largest sewers downstream of the centralized network (carrying the biggest loads in the networks with the largest diameters) were selected to showcase the implications of structural failure. To ensure the construction of redundant paths surrounding the locations of each blocked sewers for the centrality approach, we created compromise rankings as: *MIN* = σ_*i,j*_ – *c*. In this formula, the former (σ_*i, j*_) represents the normalized shortest path lengths (between 0 and 1) from all nodes *i* to the source of blocked sewers *j*, and the latter (*c*) represents the eigenvector centrality values (between 0 and 1) for all nodes. This approach (by ranking nodes from minimum to maximum values) results in higher rankings/priorities to the nodes with more eigenvector centralities (nodes (in-)directly interacting with the main collectors), and with shorter distances to the source nodes of the blocked sewers to construct the redundant paths. Therefore, this allows us to implement redundant paths from the nodes, which are both close to each blocked sewer and close to the larger pipelines.

Note that within our approach, except for the inlet offset for the sewers, other network parameters remain intact (e.g., sewer slopes and diameters), implying that the total storage volume of all centralized and decentralized networks remains constant before and after adding redundant paths. Therefore, this enables unlocking the characteristics of flow redistribution single-handedly through networks.

### 2.5 Resilience evaluation

The resilience performance of all (de)centralized USNs was evaluated using Eq. (7). The performance was assessed during low/medium rainfall event (5-year) and high-intensity event (25-year) for functional failure analysis. The design rain event (2-year) was also utilized for structural failure analysis. These rainfall events were selected based on engineering judgments and Iranian standards and regulations in flat terrains. The following hydraulic performance indicator (*HPI*) was used for this purpose in this study (Möderl et al., 2009), as shown in Eq. (7).

(7) $$HPI=100\times \left(\frac{V_{flooding}}{V_{runoff}}\right)\left(\%\right)$$

where, *HPI* represents the hydraulic performance indicator, *V_flooding_* is the total ponded flood volume [*m*^3^], and *V_runoff_* is the total runoff volume [*m*^3^]. Note that the total ponded flood volume is summation of the flooded water at all nodes that leaves and then renters the drainage network once the flood occurs (i.e., hydraulic gradient line at nodes exceeds a specific threshold).

### 2.6 Case studies

**Fig. 5:**
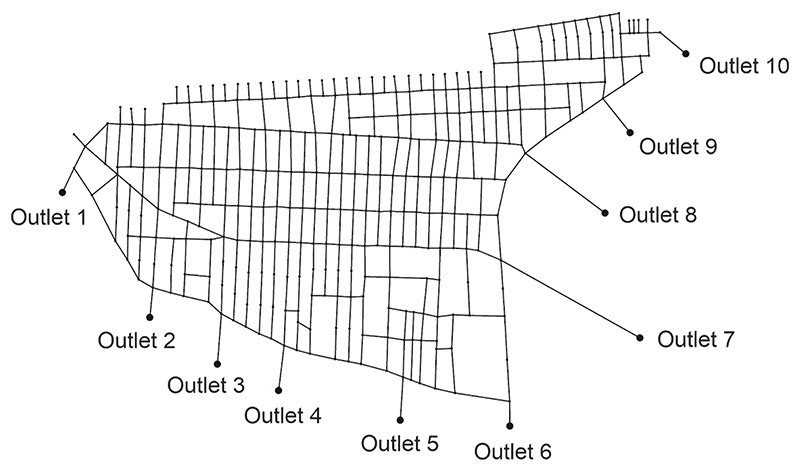
Case study (base graph): a flat area within the city of Ahvaz.

The entirely flat terrain case study (Fig. 5) is a section within a large industrial city, Ahvaz, in Iran with an area of 500 ha, 181 subcatchments, 530 sewers, and 10 outlet candidates (in the proximity of the Karun River). The city has a relatively hot and dry climate and has been recently experiencing high-intensity, short-duration rainfall events for two/three instances per year. As the city does not currently possess any stormwater management system, this has created numerous problems for authorities and citizens, such as prolonged urban flooding. Additional details regarding this case study can be found in (Bakhshipour et al., 2019).

It is important to note that the centralized and decentralized networks generated in this study with various numbers of redundant paths are published with open access on the GitHub page (https://github.com/iut-ibk/Benchmark-CaseStudies-Ahvaz.git). They can serve as infrastructure benchmarks as semi-real USNs as the produced networks are free from the constraints and boundaries often imposed by real-world systems. This can also help when facing with limited availability of real-world case studies for exploring and exploiting various models and analyses.

## 3 Results and discussion

### 3.1 Functional failure

**Fig. 6:**
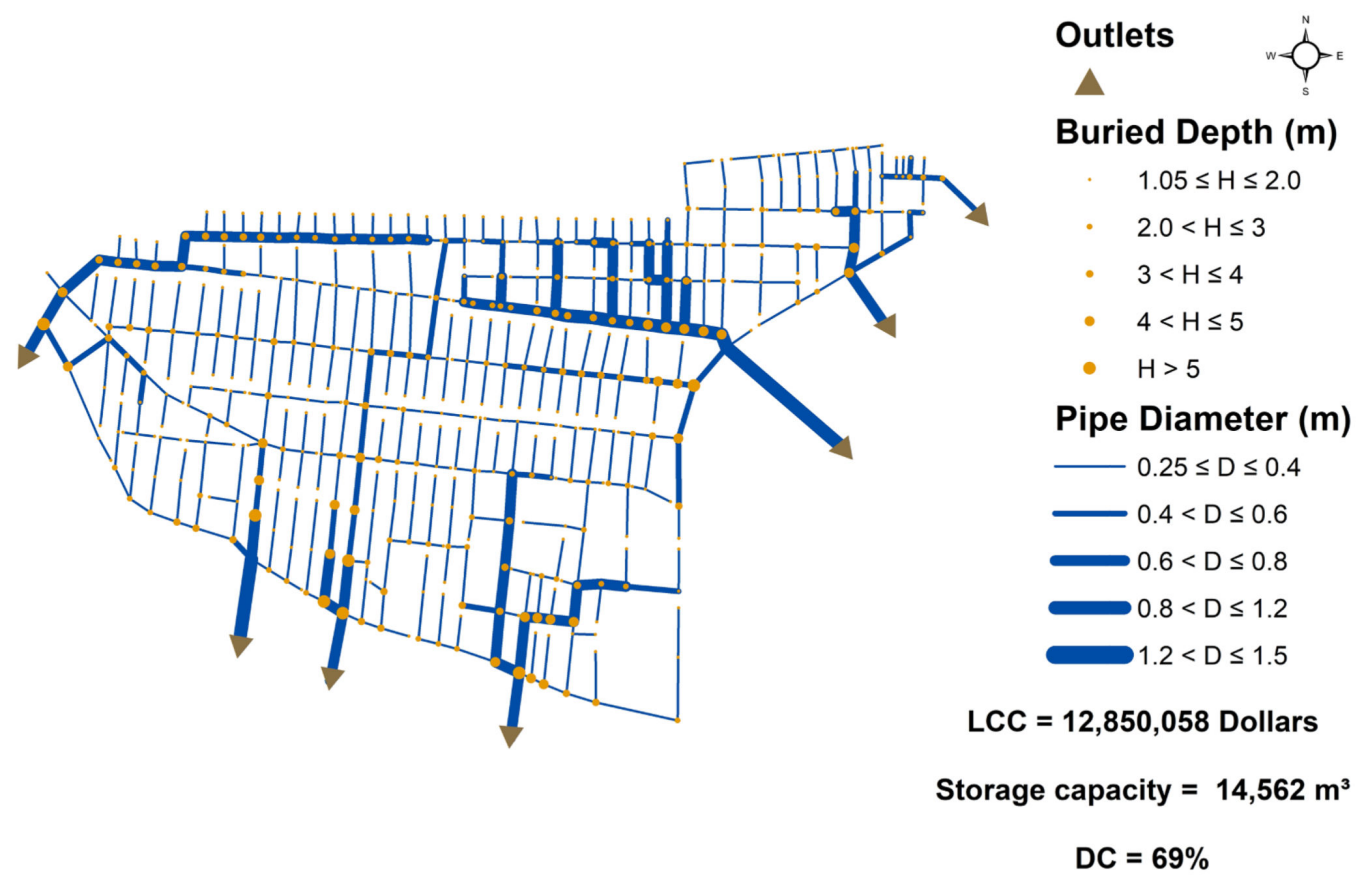
Optimal (decentralized) design with *DC* = 69%.

**Fig. 7:**
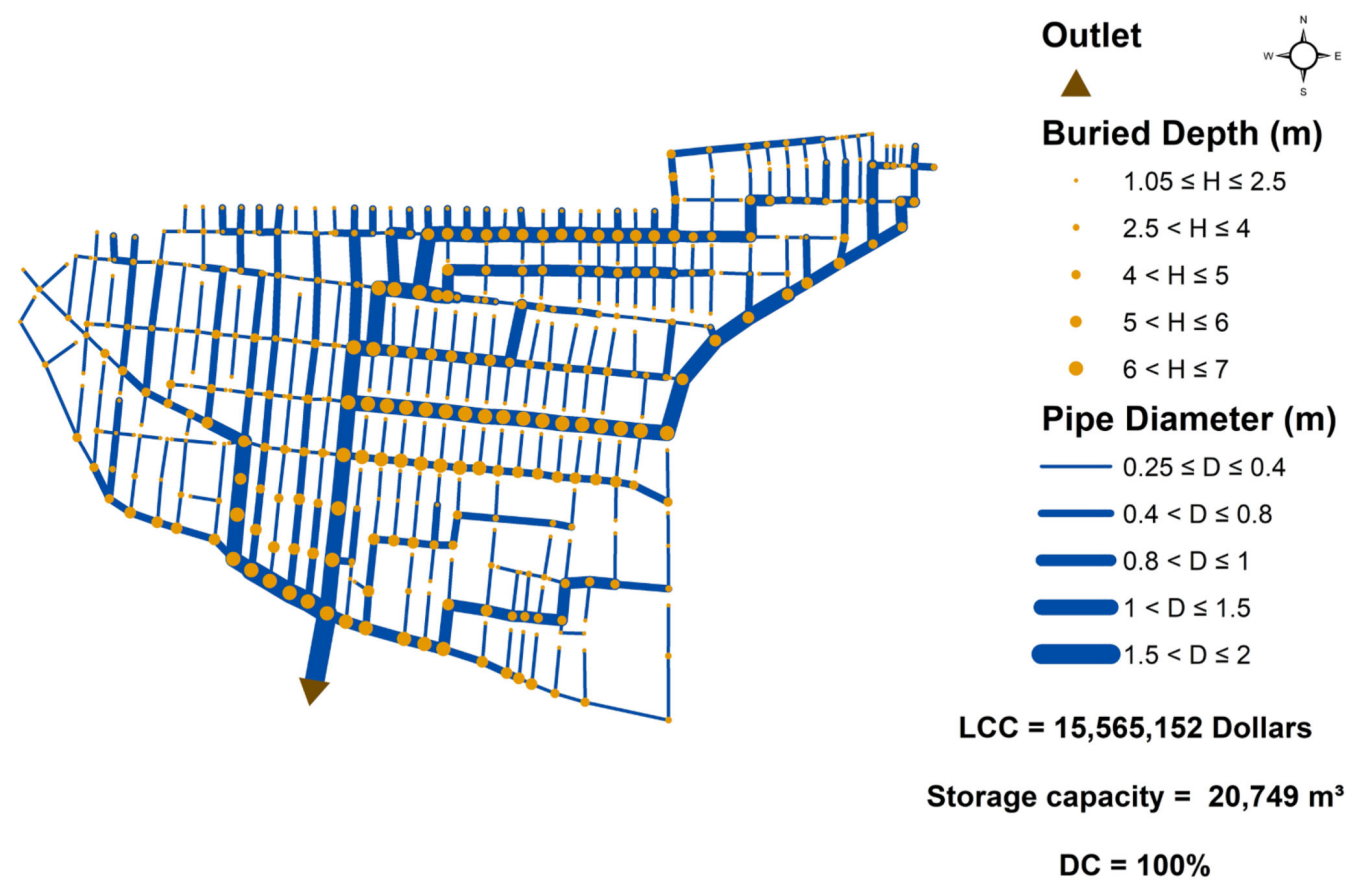
Fully centralized design with *DC* = 100%.

The optimal (decentralized) network as well as the best centralized one (among all the centralized layout configurations) were characterized as shown in Figs. 6 and 7, respectively. The *DC* for the optimal (decentralized) layout is found to be 69% (7 outlet selected out of 10 outlet candidates) as shown in Fig. 6. The decentralized network also achieved an approximately 22% lower LCC than the centralized network (with *DC*=100%). As detailed in Hesarkazzazi et al. (2022), this finding sheds light on the importance of topological decentralization during the design stage. Furthermore, total system storage capacity (i.e., the maximum capacity at which the system can accommodate volumetric flows) of centralized solution is achieved 43% larger than that of the decentralized system. The reason is correlated with the fact that components in centralized network are heavily interconnected, enforcing larger diameters during the design optimization, and therefore, bigger storage capacity. Then, these two networks were proceeded to the implementation of redundancy-promoting strategies.

**Fig. 8:**
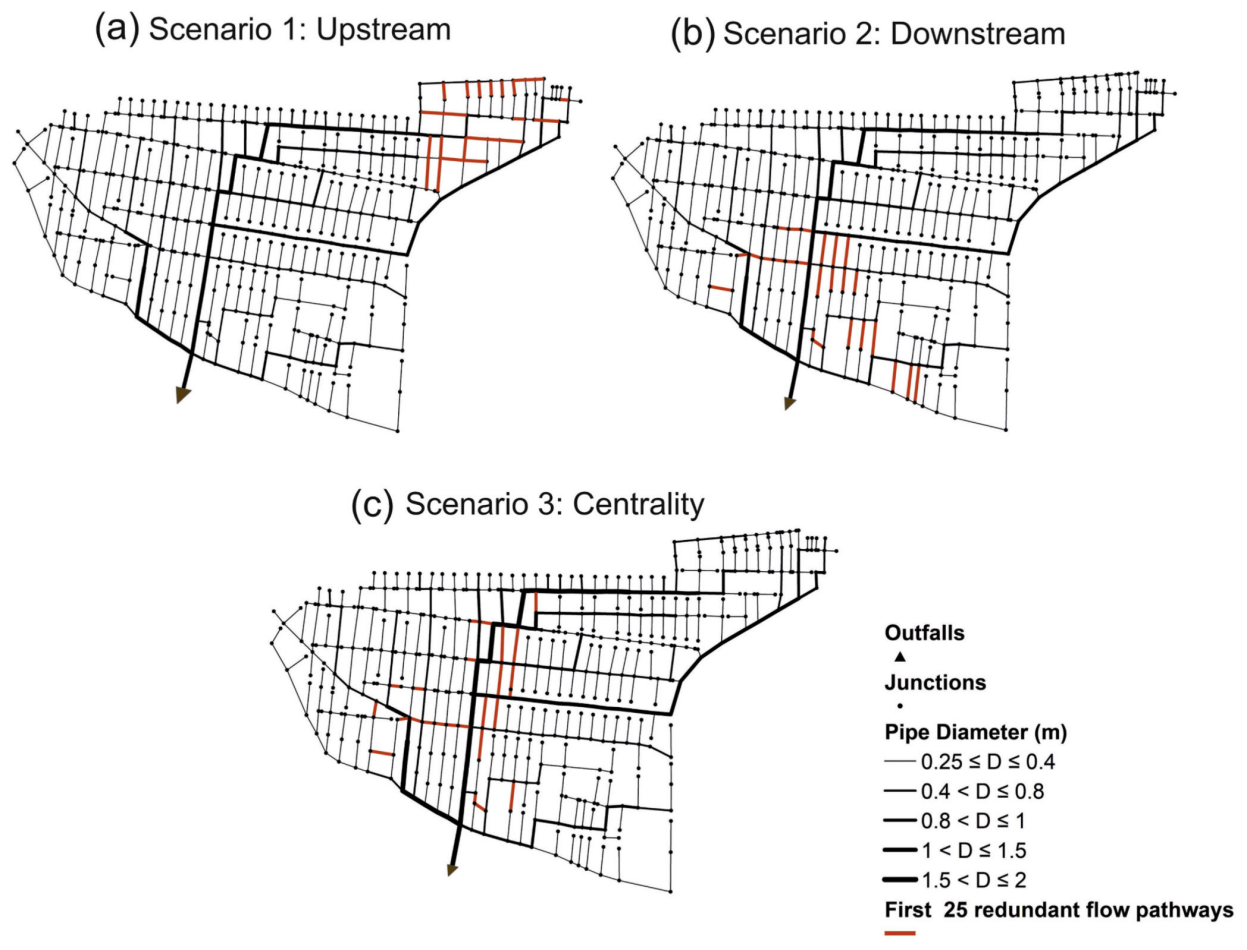
Showcasing the three redundancy scenarios defined in this study by plotting the first 25 redundant flow paths for the centralized network:(a) Scenario 1: upstream approach, (b) Scenario 2: downstream approach, (c) Scenario 3: centrality approach.

**Fig. 9:**
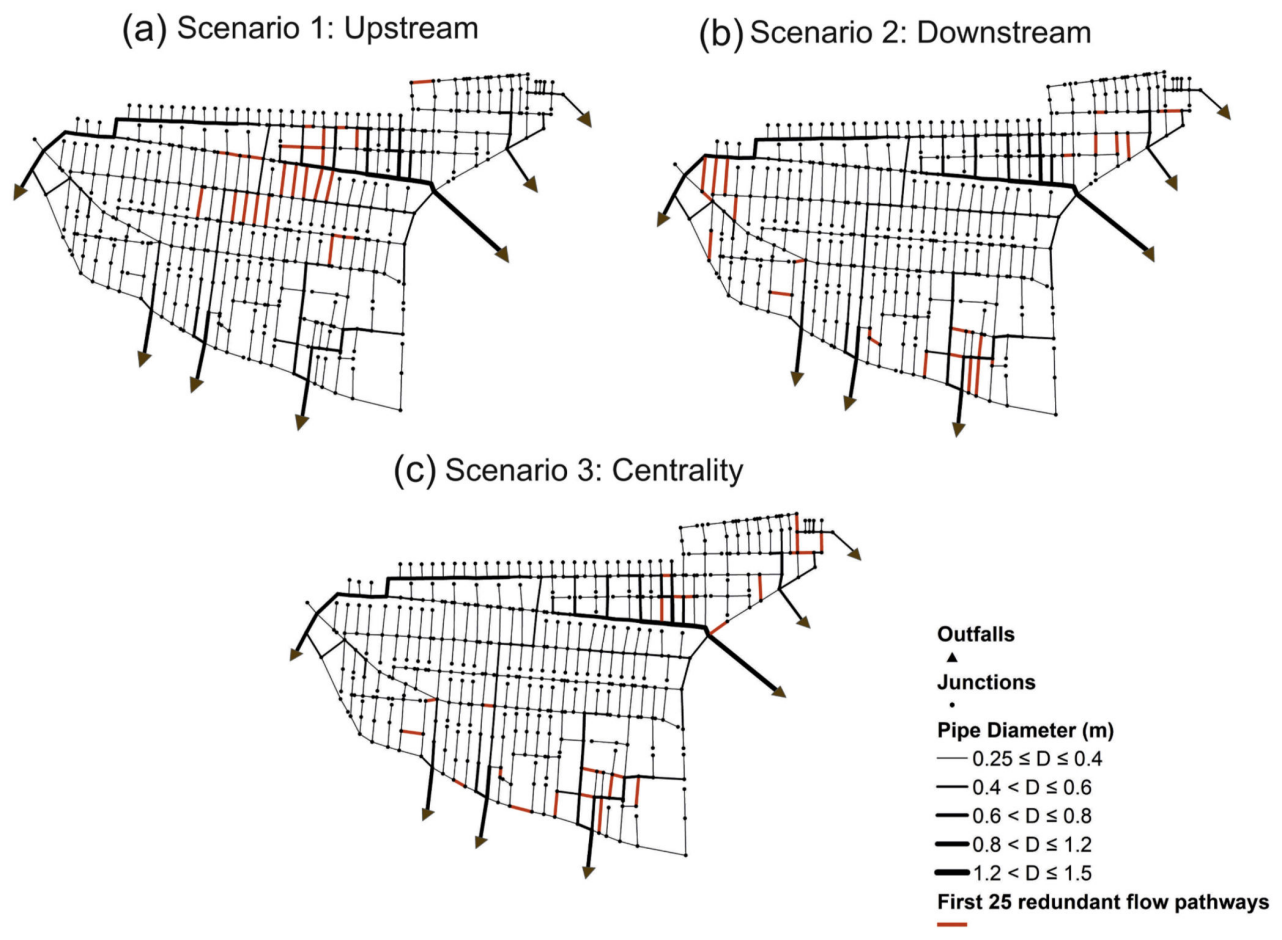
Showcasing the three redundancy scenarios defined in this study by plotting the first 25 redundant flow paths for the decentralized network:(a) Scenario 1: upstream approach, (b) Scenario 2: downstream approach, (c) Scenario 3: centrality approach.

To showcase the implementation of redundancy scenarios in this study, the first 25 redundant flow paths were allocated and shown for both centralized and decentralized networks of the case study in Figs. 8 and 9, respectively. Fig. 8(a) and (b) show that redundant paths were accurately in placed from upstream and downstream, respectively. However, as discussed before, scenario 3 (centrality approach) seeks to start targeting the nodes whose connections to their adjacent nodes interact with the primary pipelines of the network. This can be seen in Fig. 8(c) where the first 25 redundant paths were constructed mainly around the main central collector of the network.

The same illustration of the three redundancy scenarios outlined above has been also demonstrated for the decentralized network in Fig. 9. It is evident that the introduction of additional pipes was successfully undertaken at upstream and downstream sections of the network, as shown in Fig. 9(a) and (b), respectively. Further, scenario 3 (centrality approach) has been able to successfully construct the redundant paths interacting with the large sewer diameters, as shown in Fig. 9(c). Note that in this study, the determination of upstream, downstream and centrality-based locations was performed globally in the decentralized networks. This implies that we may have a situation where the redundant paths were disproportionally distributed in some specific branches/subgraphs of the decentralized network than the other branches. Therefore, in the future, the proposed framework could also include the subgraphs of the decentralized network to investigate the effect of homogenously distributing the redundant paths in each branch (subgraph) of the network.

**Fig. 10:**
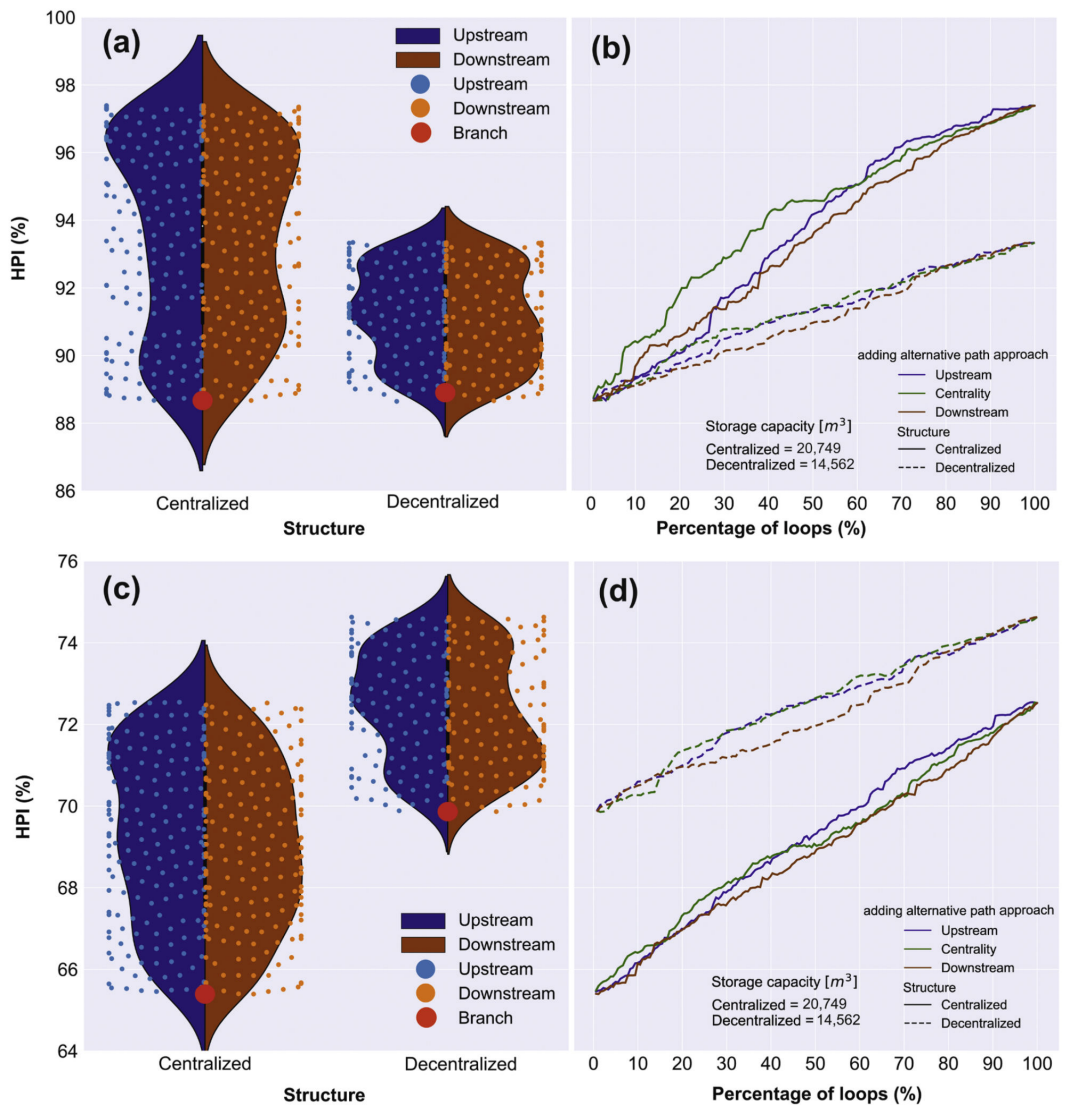
Resilience performance (HPI measure) of centralized and decentralized networks while redundancy (alternative water flow paths) is added using the upstream, downstream and centrality approaches. Each dot in Fig. 10(a) and (c) represents an HPI value corresponding to a network with various numbers of redundant paths. Fig 10(a) and (b) are the performances during the 5-year rain event, and Fig. 10(c) and (d) are the performances during the 25-year rainevent.

After the network characterization, the impact of redundancy implementation on the two designed structures (centralized with *DC*=100% vs. decentralized with *DC*=69%) is investigated via resilience assessment (HPI measure) subjected to two types of rainfall-induced functional failure (5- and 25-year storm events with total rain amounts equal to 32.3 mm and 49.9 mm, respectively). These results are shown in Fig. 10. Based on our case study, total number of 181 redundant flow paths were introduced to the designed tree-like centralized network, while 131 redundant pathways (after pre-selection) were subsequently added to the optimal tree-like decentralized one to avoid compromising its decentralized structure. Then, we normalize the number of redundant flow paths (divide by the total number of redundant paths for each structure) to yield 0-100% loop percentage for both structures (see Fig. 10(b) and (d)).

Fig. 10(a) and (b) indicate the resilience performance of all networks (i.e., from a fully branch network to fully looped one) for two structures (i.e., centralized and decentralized) under the 5-year rainfall event. Fig. 10(a) shows the swarm plot (as a type of scatter plot) of HPI values on top of the violin plot (depicting the distribution of HPI values (Hintze and Nelson, 1998)) with respect to the two structures and their relationship with upstream (blue colour) and downstream (orange colour) approaches along with the results from the branch network. Each dot in Fig. 10(a) represents a HPI value corresponding to a network with various numbers of redundant paths. Fig. 10(b) shows the HPI values plotted against the percentage of loops, alongside the implication of centrality strategy.

As shown in Fig. 10(a), the HPI values for the branch/tree-like structures were approximately 89% for both centralized and decentralized layouts. The introduction of extra paths from upstream and downstream locations until the placement of the last one (100% loops) enhanced resilience by 8% for the centralized layout and 4% for the decentralized layout. It is also apparent from Fig. 10(b) that introducing redundancy to the centralized layout gained more momentum for resilience growth than the decentralized layout. This is because in decentralized networks, volumetric flows can be discharged through several separated main collectors, as opposed to centralized ones where there exist very limited primary collectors. Therefore, the lack of effective flow distribution pertaining to centralized networks can, to some extent, be compensated via redundant path implementation. Combining this effect with a substantial storage capacity in the centralized system (approximately 43% bigger than that of the decentralized system) forms a recipe for more rapid resilience growth. It is evident that except for the first 20-25% of loops for the centralized solutions, the placement of additional loops at upstream outperformed those downstream for both structures. This indicates that the placement of initial redundant pathways (in this case up to the first 25%) both at downstream and upstream performs quite similarly, sometimes in favour of downstream. Further, the performance of all three approaches tends to converge for decentralized scenarios when passing almost 80% loops. It was also found that the centrality approach substantially out-performed the other two strategies by up to 60% loops for centralized and 70% loops for decentralized configurations. Thereafter, the up-stream approach performed slightly better than the centrality one. This is because despite the improved performance by first targeting the nodes for connection to the main collectors by the centrality approach, placing around 65% of the loops all at upstream could attenuate flood flows more effectively. This attenuation is induced by increasing the time of concentration of the entire catchment and dispersing the increased discharges more homogenously through the network.

Similarly, Fig. 10(c) and (d) represent the same implications as Fig. 10(a) and (b) but when the networks are subject to high-intensity rainfall event (25-year event). As shown in these figures, the significance of decentralization implemented during the design stage is more highlighted under such extreme storm condition. Similar to the 5-year event, the resilience growth rate of centralized scenarios (ranging by 8%) was recorded to be greater than that of decentralized ones (ranging by 5%). However, the decentralized branch/tree solution recorded an HPI of 70% (as opposed to 65% for the centralized one), continuously outperforming the centralized solutions. This finding indicates that despite imposing good flow redistribution via redundancy on centralized solutions alongside their substantial storage capacity, topological decentralization executed at the design stage plays a more crucial role in achieving a higher resilience under heavy rain events. In terms of different redundancy deployment strategies, the patterns achieved are somewhat analogous to those obtained under the 5-year event. This shows that the upstream approach outperformed the downstream one, starting from 25% for centralized and 20% loops for decentralized solutions. However, the implementation of these three approaches tends to perform relatively similar for the decentralized network passing around 80% loops. Moreover, centrality approach outperformed the other two approaches by up to 45% loops for the centralized solutions, whereas its performance was relatively close to the upstream approach in decentralized networks. These implications, combined with those achieved under the 5-year rainfall event, indicate that the more extreme the event, the more significant the impact of redundant paths placement at upstream.

**Fig. 11:**
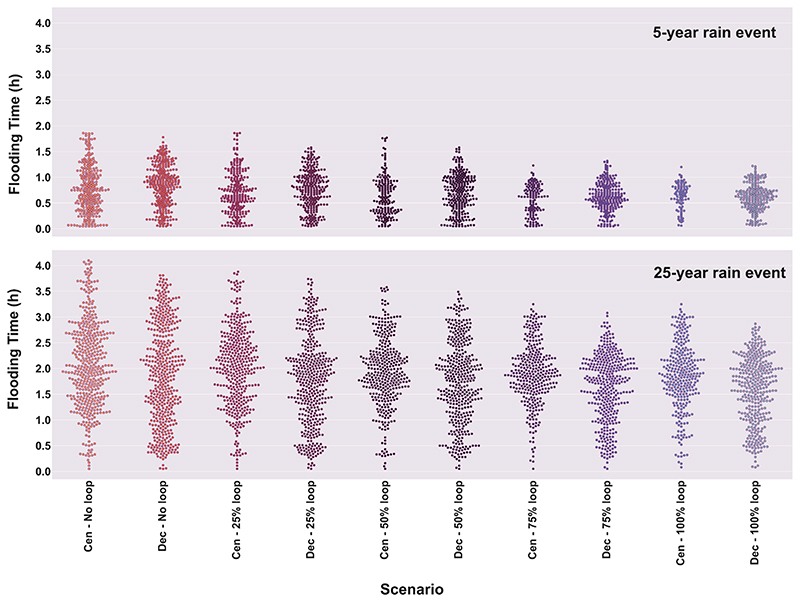
Resilience performance (via flood duration) during the 5- and 25-year rain events for 0 (branch), 25, 50, 75 and 100% loop percentages (implemented by centrality approach) for both centralized (Cen) and decentralized (Dec) structures. Each dot represents a flood duration value corresponding to a network with specific percentage of loops shown in x-axis.

Furthermore, as this region (entirely flat) often faces a problematic inundation period, we plotted below the flood duration in hours for both structures with no loops, with 25% loops, 50% loops, 75% loops and 100% loops (maximum) as shown in Fig. 11 under 5- and 25-year rainfall events. Note that as the centrality approach generally out-performed the other two approaches (i.e., upstream and downstream), we only showed the flood duration results when redundant paths were constructed using the centrality approach (Fig. 11). The implications of the other two approaches can be found in this manuscript’s Supplementary Material.

Fig. 11 shows that, as a general pattern, when more redundancy is introduced to both structure types, the observed flood duration is more reduced. The flood duration was reduced from approximately 2 h for the branch network to approximately 1.25 h for the 100% looped system under the 5-year storm event for both structures. Likewise, flood duration gradually waned under the 25-year event when redundancy was introduced with each step of implementation (25% loops, 50% loops, etc.), ranging from more than 4 h for the branch network to approximately 3 h for the fully looped structure. In addition, the decentralized layouts yielded a shorter flood duration than their centralized counterparts with the same loop percentage. However, there are two exceptions for the networks with 75% and 100% loops under the 5-year storm even, where the centralized solutions exhibit lesser flood duration compared to their decentralized ones. The reason is that when combining a big storage capacity pertaining to centralized solutions (43% larger) with high level of redundancy (around 75% loops and more), centralized networks outperform decentralized in terms of flood duration. However, under heavier storm event (25-year event) such trade-offs turn in favou of flow redistribution (imposed by redundancy) rather than storage capacity, and that is why decentralized structures outperform centralized ones under heavier events in terms of both flood magnitude and duration, regardless of the (big) capacity in the centralized solutions. These implications further support the effectiveness of redundant paths for reducing flooding duration as another major component of the resilience definition.

### 3.2 Structural failure

**Fig. 12:**
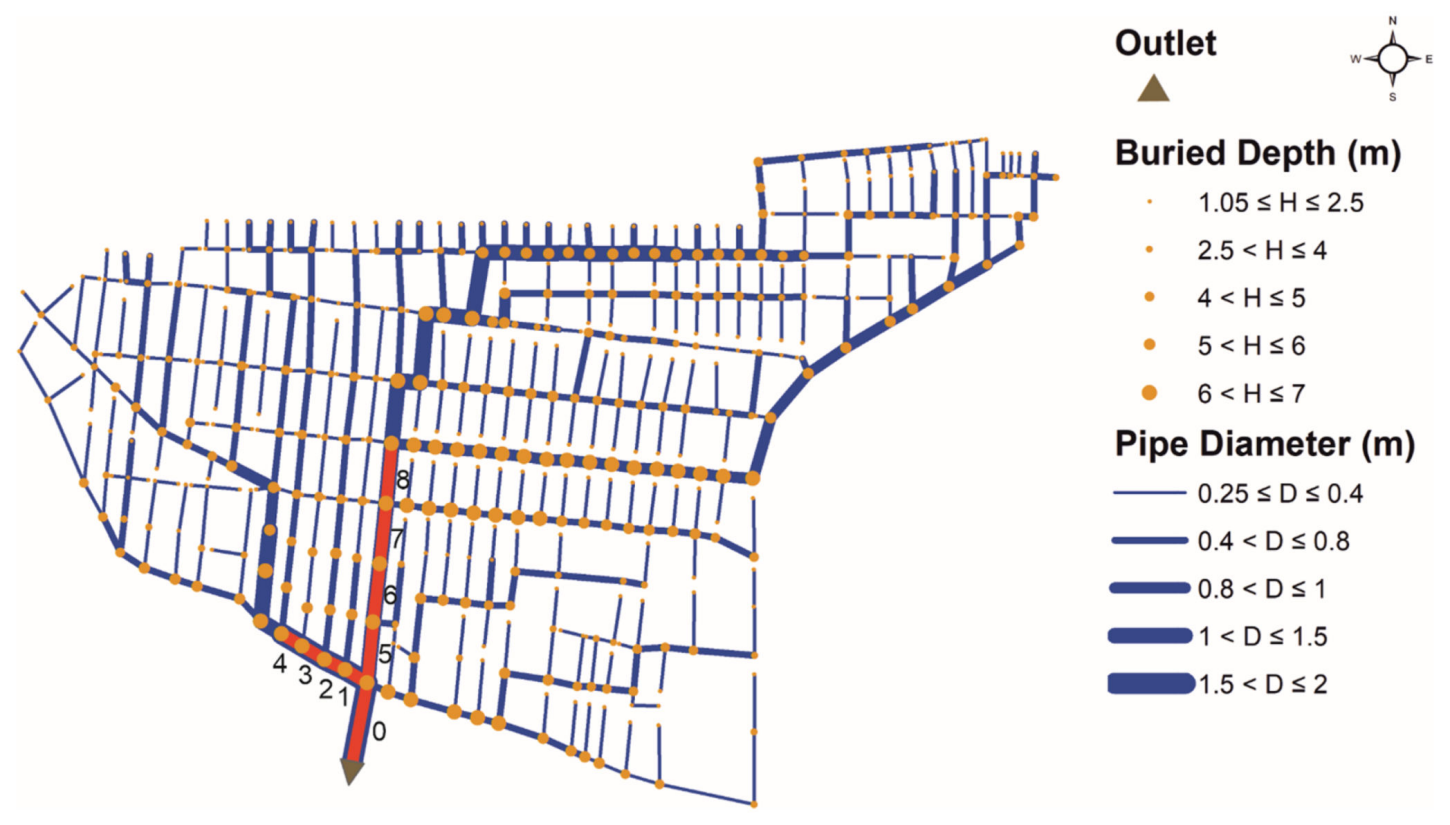
Fully centralized design solution along with the nine largest sewers at downstream for the purpose of structural failure.

With regard to structural failure, we significantly reduced the relevant sewer diameters to 10 mm to replicate the sewer blockage in the system (Mugume et al., 2014). Nonetheless, as opposed to the method introduced in Mugume et al. (2015), where sewer collapse is simulated randomly and cumulatively (which can be more useful when the system experiences earthquake stress or random attacks), we addressed sewer failure successively and only for large sewers in this study. This is because the failure of upstream-located sewers did not significantly deteriorate the system resilience as they often carried lower discharge volumes. Moreover, the decentralized scenarios were disregarded herein for the purpose of structural failure because the centralized layout is more vulnerable due to the existence of unique and limited pathways (major collectors) to discharge the design peak runoff. As a result, to better showcase and support the implication of blockage consequences on resilience, only the nine largest sewers downstream were investigated (see Fig. 12).

**Fig. 13:**
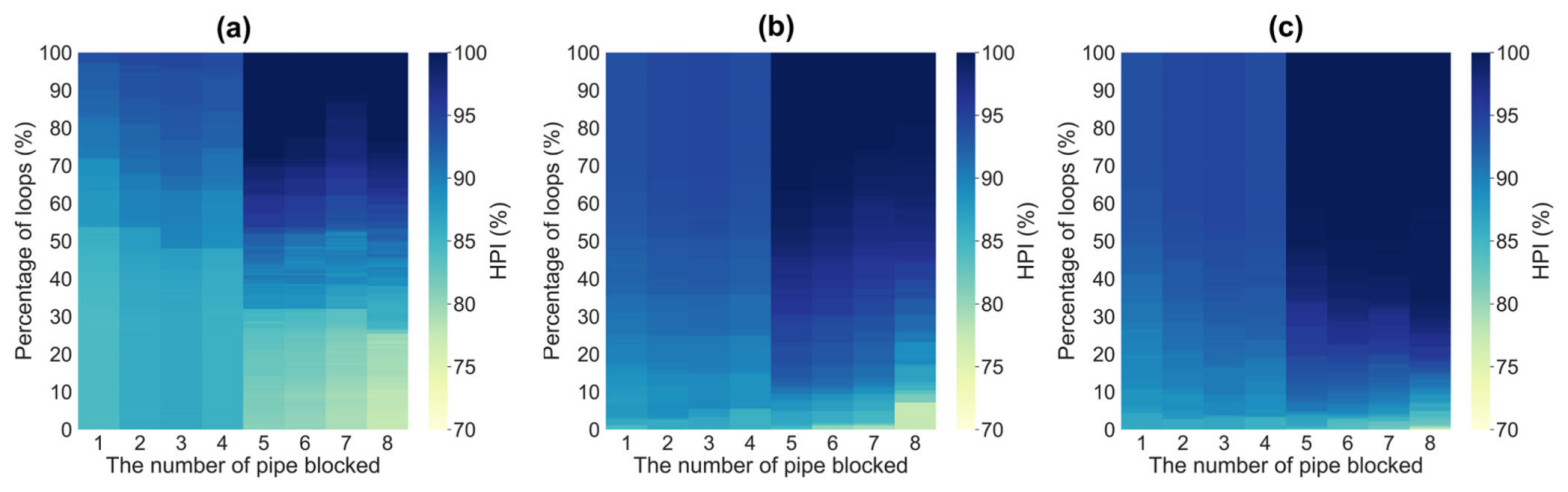
Resilience performance (HPI measure) of the fully centralized network with respect to the failure of eight largest sewers at downstream (except for the sewer number 0, which is connected to the outlet) under design rain event; wherein (a) indicates upstream approach, (b) indicates downstream approach (c) indicates modified centrality approach.

The results indicate that the blockage of sewer 0 (directly connected to the outlet) would completely fail the entire system even under the design rainfall event. This highlights the vulnerability of centralized layouts since the design peak runoff cannot escape from the system. The resilience results of the other eight largest sewer failures under design rainfall are depicted in Fig. 13(a)-(c). Fig. 13(a) demonstrates the results corresponding to the upstream approach, (b) demonstrates the results corresponding to the downstream approach, and (c) demonstrates the results corresponding the centrality approach with a minor modification explained in Section 2.4. As shown in Fig. 13(a), cumulatively adding alternative water pathways at each step (i.e., 10% loops, 20% loops, etc.) with upstream approach could not significantly enhance the structural resilience (via HPI measure) under the occurrence of sewer failures 1-4 (these sewers had the largest diameter, that is, 2 m). In other words, resilience was just slightly improved after implementing each loop step when cumulatively introduced from the upstream locations. Such an implementation, however, is accompanied by a better improvement when considering the failure of sewers 5 to 8, thanks to their central locations in the network. Once the cumulative implementation of redundancy was initiated by downstream approach, the resilience was enhanced more rapidly at each step under all sewer failure scenarios (see Fig. 13(a) and (b)). This is associated with placing redundant paths in the same sections of failure locations (i.e., both failure and redundancy locations are at downstream sections) for effectively storing and dispersing the backwater coming from the blocked sewers. This resilience enhancement is specifically more apparent for the failure of sewers 5 to 8 relative to the upstream approach. This is again related to the position of the sewers located in the center/middle portion of the network. To place this into context, as the backwater resulting from these blocked sewers travelled upstream, alternative pathways were able to retrieve and exchange the discharges to the other parts of the network, transferring them between the main/large collectors for better and quicker dispersion of the flows.

In addition, once the redundant paths were implemented using the centrality approach, the resilience improvement rate received even more successful momentum compared to the downstream approach (see Fig. 13(b) and (c)). Centrality approach marginally outperformed the downstream one under the failure of sewers 1-4. Again, this is because the backwater effect occurring from these sewers does not pass main/large collectors along their journey to the upstream areas (upper left of the network), to help them in retrieving a portion of this water. In contrast, a more significant resilience increase was observed after the collapse of sewers 4-8 (specifically sewer 8) compared to the down-stream approach. For instance, the failure of sewer 8 had a constant HPI value of 75% from the network without loop up to the first 10% loops via the downstream approach. Whereas HPI measure concluded the first 10% loops at approximately 87% using the centrality approach. This is correlated with the initiation of constructing the extra pathways surrounding each blocked sewer via the modified centrality approach, by leveraging the flow redistribution of backwater flows, and thereby, yielding a better and quicker flood attenuation. From Figs. 12 and 13(c), it is apparent that the backwater coming from the failure of sewer 8 would be more accessible to the major collectors/pipelines surrounding its location. Thus, placing alternative paths first around those critical locations using the centrality approach may further help in retrieving and exchanging the design flow volumes between such major pipelines.

## 4 Limitations, advantages, and future work

As the implications of this study are based on one specific case study and the local precipitation properties, the broad generalization of the results may not be always advisable. However, we believe that the patterns achieved for both centralized and decentralized layouts under functional and structural failure would still be relevant and applicable, regardless of the storm and the case study characteristics. It should also be noted that we did not change the major structural characteristics of centralized and decentralized networks via redundancy (that is, diameter, slope, length, and total storage capacity remained constant for each structure before and after loops introduction). However, with this minor modification (creating redundancy/redundant flow paths), we could push up resilience performance even by up to 8% under functional failure. Enlarging added sewers can also be considered at critical locations for further resilience enhancement. Note that depending on the phase of project (e.g., design or rehabilitation stage), introducing redundancy can change the costs related to the system (e.g., LCC). For instance, during the planning stage, introduction of redundant paths converts the existence of two manholes to one (see Fig. 3), and thus, could reduce the overall design costs. However, regardless of the project phase, based on our previous experimentation and LCC formula used in this study, expenses related to manholes are much smaller than piping process in the network. Hence, although, we did not discuss the implications of redundancy implementation for LCC variations, redundancy in our context would have marginally affected the LCC. In the future, we aim to use cumulative runoff areas of each pipe as the edge weighting function (instead of diameters) for ranking the critical nodes determined by (eigenvector) centrality approach. This allows identifying and ranking the influential nodes more accurately for the introduction of redundant flow pathways. We will also present the transition towards an integrated framework by introducing limited alternative path flows to the most sensitive locations of (de)centralized networks. This is achieved by optimizing the inlet offset of the added sewers while integrating the green-blue infrastructures (GBIs). This framework will be implemented based on the weak points of the network or even considering functional interdependency (e.g., existing a hospital or school over the road network) to successfully avoid or minimize any functional and structural failure surrounding these critical infrastructures.

## 5 Conclusion and final remarks

There is an urgent need to transition towards resilient USNs at all stages, as climate-change-driven precipitations, sewer blockages, and aging elements are deteriorating the functional and structural operation of these systems. Therefore, a redundancy-promoting framework was developed in this study based on three approaches: cumulatively introducing redundant water flow paths from upstream, downstream and sensitive locations. This framework seeks to enhance the USN resilience under functional and structural failure for optimal centralized and decentralized USNs. This was achieved by leveraging an efficient (re)distribution of water flows throughout the network. The main findings of this study are as follows:

- The importance of topological decentralization is highlighted during the design stage in terms of both capital savings and resilience enhancement.
- Using a tailored graph-theory-based topological measure (i.e., eigenvector centrality), the sensitive and critical locations for introducing redundant paths are identified by first interacting with the main collectors of the network.
- Constructing redundant paths (forming loops) initially at critical locations (using eigenvector centrality approach), outperforms the other two strategies experiencing functional failure. However, after exceeding a loop threshold, the placement of redundant paths all at upstream performs marginally better (especially under heavier storm events).
- Redundancy in centralized networks contributes more to resilience than that in decentralized networks. This is because the lack of efficient flow distribution in centralized networks can be compensated to certain extent via redundant path implementation. Combining this effect with their substantial storage capacity (compared to decentralized ones), forms a recipe for more rapid resilience growth.
- Although the resilience growth of centralized networks with redundant flow paths (forming loops) progresses more rapidly than decentralized solutions, the decentralized layout solutions with loops continuously outperform centralized ones during heavy rain events.
- Introducing redundant paths at downstream would be more promising for centralized layouts experiencing structural failure because the blockage in major pipelines located downstream (with more discharge volumes) would affect the system’s resilience more than upstream blockages.

## Supplementary Material

Supplementary material associated with this article can be found, in the online version, at doi:10.1016/j.watres.2022.118910.

Supplementary Material

## Figures and Tables

**Table 1:** Case study characteristics.

Total number of junctions	Total number of sewers	Total number of Subcatchment	Total lengths (m)	Average sewer lengths (m)	Total area (ha)	Total runoff area (ha)	Total number of outlet candidates	Elevation (m)
340	530	181	75,000	67.92	500	382	10	18

## Data Availability

All networks, including the base graph, purely centralized, and optimal decentralized networks for both steep and flat terrains are available in GitHub (https://github.com/iut-ibk/Benchmark-CaseStudies-Ahvaz.git).
