# Supplementary material for: Battle of centralized and decentralized urban stormwater networks: From redundancy perspective
Source: Water Res. Author manuscript; Available in PMC 2024 Dec 5. (PMC7616898; doi:10.1016/j.watres.2022.118910)
Supplement: Supplementary Material [file EMS155467-supplement-Supplementary_Material.docx]

Supplementary Materials for

**Battle of Centralized and Decentralized Urban Stormwater Networks: From Redundancy Perspective**

Sina Hesarkazzazi^a^, Amin E. Bakhshipour^b^, Mohsen Hajibabaei^a^, Ulrich Dittmer^b^, Ali Haghighi^c^, Robert Sitzenfrei^a,^*

*^a^* Unit of environmental engineering, Institute of Infrastructure, University of Innsbruck, 6020, Innsbruck, Austria

^b^ Department of civil Engineering, Institute for Urban Water Management, Technical University Kaiserslautern, 67663, Kaiserslautern, Germany

^c^ Faculty of civil engineering and architecture, Shahid Chamran University of Ahvaz, 61357831351, Ahvaz, Iran

*Corresponding author: [*Robert.Sitzenfrei@uibk.ac.at*](mailto:Robert.Sitzenfrei@uibk.ac.at)

**Cost function and Design constraints for the case study:**

**Table ‎S.1:** Design constraints for the flat case study (Bakhshipour et al. 2019)

| Description | Constraint |
| --- | --- |
| Maximum Velocity | 4.0 m/s |
| Maximum excavation depth | 5.0 m |
| Minimum cover depth | 1.2 m |
| Minimum slope | 0.0041 if D=200 mm  0.0033 if D=250 mm  0.0027 if D=350 mm  0.0020 if D=400 mm  0.0016 if D=500 mm  0.0014 if D=630 mm  0.0010 if D=800 mm  0.0010 if D$\geq$1000 mm |

The $LCC$ evaluates the capital costs and the operation and maintenance ($O\&M$) costs of the pipe network over a typical service period of 30 years. The $LCC$ of each alternative is calculated by compiling all the capital and $O\&M$ costs using Equation S.1 to S.3 to present-day. The inflation rate of $O\&M$ cost in Iran is considered as 12% and the discount rate of the total $LCC$ as 15%. 10% of capital costs, from Iranian manual, are considered for annual $O\&M$.

|  | $LCC={Capital}_{sewer network}+{PV}_{O\&M sewer network}$ | (S.1) | |
| --- | --- | --- | --- |
|  | ${PV}_{O\&M}=\sum_{n=1}^{30} \mathrm{Annual}_{O\&M}\frac{{(1+r)}^{n}}{\left( 1+i \right)^{n}}$ | (S.2) |  |

${PV}_{O\&M}$ is the 30-year $LCC$ for $O\&M$of the sewer network, $i$ is the discount rate, $r$ is the inflation rate, and $n$ is the years of service.

|  | ${Capital}_{sewer network}=\sum_{i=1}^{NP} {CP}_{i}+\sum_{i=1}^{NM} {CM}_{i}$ | (S.3) |
| --- | --- | --- |

in which $CP$ and $CM$ represent, respectively, the construction cost of sewers and manholes. These costs are estimated as a function of pipe diameter and buried depth using Table ‎S.2. $NP$ and $NM$ represent the number of pipes and number of manholes, respectively.

**Table *S.2*:** Cost function of the case study

| Diameter  (mm) | Cost of pipes  (Million Rial/m) | Cost of manholes  (Million Rial) |
| --- | --- | --- |
| 200 | 3.50H - 2.29 | 29.95H + 54.91 |
| 250 | 3.50H - 2.21 | 33.70H + 58.66 |
| 350 | 3.38H - 1.31 | 42.03H + 62.21 |
| 400 | 3.57H - 0.91 | 45.66H + 66.46 |
| 500 | 3.62H + 0.05 | 52.85H + 75.37 |
| 630 | 3.85H + 1.57 | 56.39H + 80.05 |
| 800 | 4.31H + 3.86 | 59.90H + 84.86 |
| 1000 | 4.65H + 8.12 | 73.65H + 105.56 |
| 1200 | 5.11H + 10.84 | 79.40H + 113.64 |
| 1500 | 5.73H + 15.55 | 91.20H + 129.86 |
| 2000 | 6.78H + 23.37 | 110.91H + 159.32 |

Note: H = average buried depth

**Flood duration for the case study:**

**
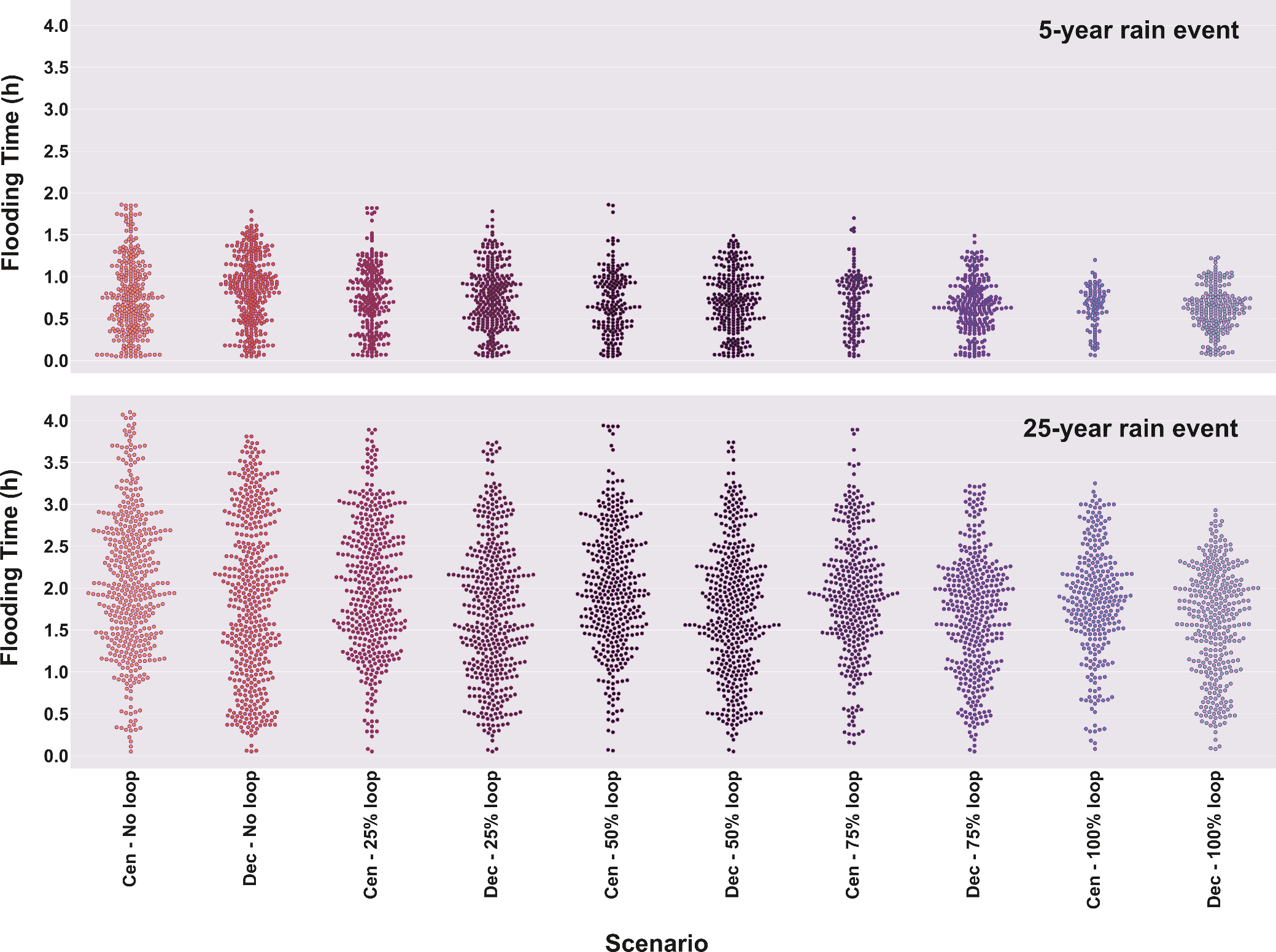
**

Figure S.1. Flooding duration for the case study when loops are constructed using upstream approach.


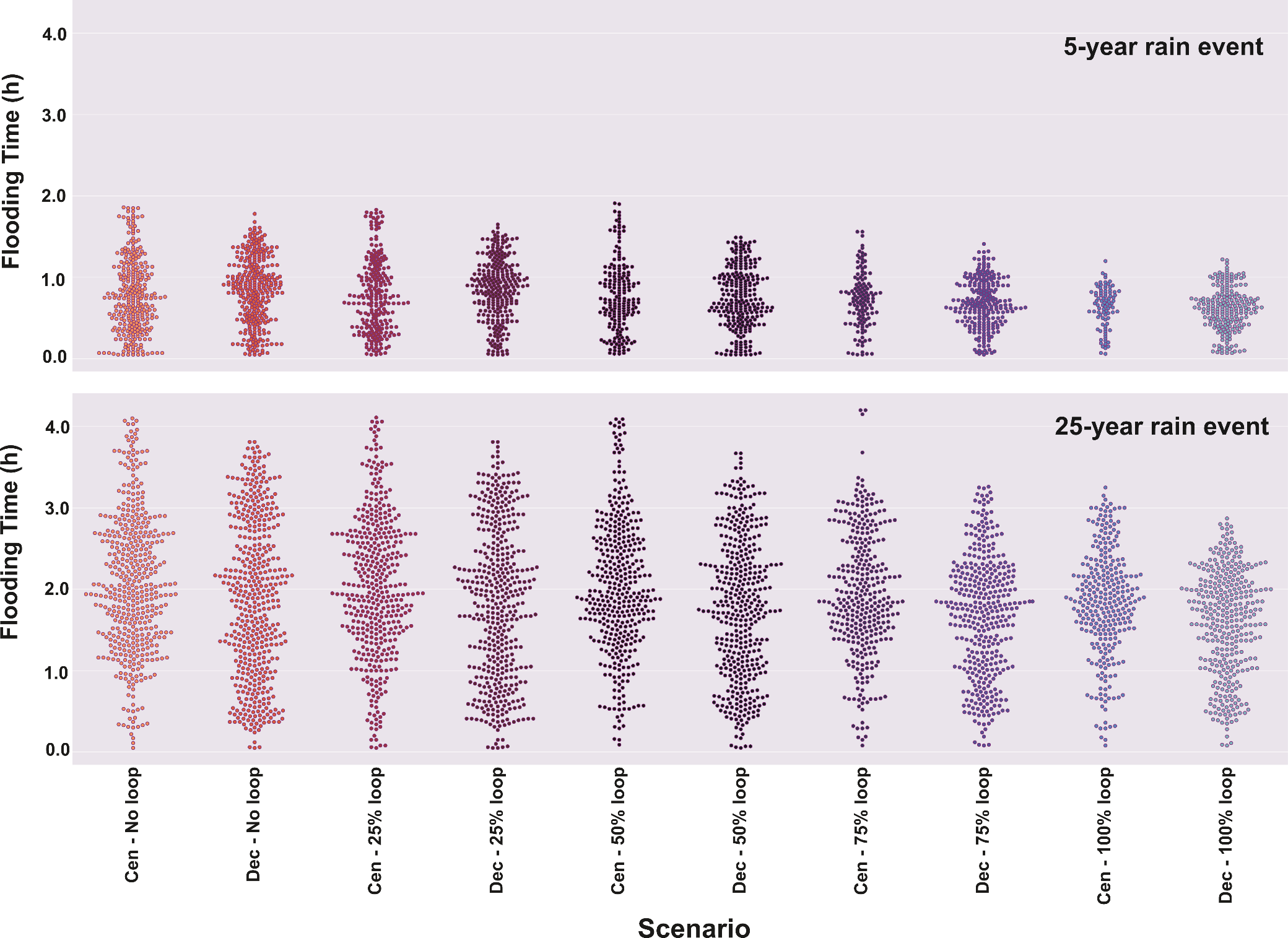


Figure S.2. Flooding duration for the case study when loops are constructed using downstream approach.

**References**

Bakhshipour, Amin E.; Bakhshizadeh, Milad; Dittmer, Ulrich; Haghighi, Ali; Nowak, Wolfgang (2019): Hanging Gardens Algorithm to Generate Decentralized Layouts for the Optimization of Urban Drainage Systems. In J. Water Resour. Plann. Manage. DOI: 10.1061/(ASCE)WR.1943-5452.0001103.
